# Role of Ultrasound in Tracheotomy and Cricothyrotomy: A Research Study of Midline Cervical Vasculature in Healthy Individuals

**DOI:** 10.7759/cureus.92711

**Published:** 2025-09-19

**Authors:** Okechukwu Nkwocha, Chinedu J Enwerem, Michael Adeniyi, Tarek Alambrouk, Unaiza Jawad, Sadia Javed, Haider Hilal

**Affiliations:** 1 School of Medicine, St. George's University School of Medicine, Newcastle upon Tyne, GBR

**Keywords:** airway management, midline cervical vasculature, pocus, tracheotomy, ultrasound

## Abstract

Introduction

Tracheotomy and cricothyrotomy are life-saving airway interventions that carry a risk due to the presence of midline vascular structures in the anterior neck. Injury to these structures during blind landmark-based procedures can lead to serious complications. Ultrasound (US) is a non-invasive, real-time imaging modality that enables direct visualization of cervical anatomy and vascular variants. Despite its advantages, its routine use is not yet standardized in clinical protocols. This study aims to evaluate the prevalence of midline vasculature using US in healthy individuals and to review current guidelines regarding US use in airway access. The aim of the study is to assess the prevalence of midline cervical vasculature using US in healthy adults and to review national and international guidelines on US use in tracheotomy and cricothyrotomy procedures.

Methods

This cross-sectional observational study included 80 healthy volunteers in the age range of 18 to 49 years. High-resolution US scans were performed at three anatomical levels: the inferior border of the thyroid cartilage, the superior border of the cricoid cartilage, and the inferior edge of the thyroid isthmus. The presence of midline vascular structures was recorded. A concurrent review of airway management guidelines (American Society of Anesthesiologists, Difficult Airway Society, Royal College of Anaesthetists, and others) was also undertaken.

Results

Midline vascular structures were identified in 23 participants (28.7%). At specific anatomical levels, 18 participants (22.5%) had vessels at the thyroid isthmus, three participants (3.8%) had at the cricoid cartilage, and two participants (2.5%) had at the thyroid cartilage. No significant association was found between vascular presence and demographic factors such as sex, BMI, or ethnicity, with a p-value of 0.6 (Gender) and 0.9 (Ethnicity). Guideline review revealed no consistent recommendations for routine US use in tracheotomy or cricothyrotomy.

Conclusion

The US detected significant vascular structures in the midline neck, particularly at the thyroid isthmus level, in a substantial portion of healthy individuals. These findings support integrating the US into standard airway management protocols to enhance safety and accuracy.

## Introduction

Tracheotomy, the surgical creation of an airway opening in the trachea, is one of the oldest known surgical procedures, with origins dating back to ancient medical texts [1]. Early descriptions resembling tracheotomy appear in documents such as the Ebers Papyrus (circa 1550 BC) and the Sushruta Samhita (circa 600 BC), underscoring the longstanding recognition of securing the airway in emergencies [1]. Over centuries, the procedure evolved through the works of Greek and Renaissance physicians, including Antyllus in the 2nd century AD and Antonio Musa Brasavola in the 16th century, who performed the first documented successful tracheotomy [2]. These developments laid the foundation for modern airway management techniques.

Today, tracheotomy and cricothyrotomy remain vital, life-saving interventions used to bypass upper airway obstruction in both elective and emergency settings [3]. However, these procedures carry risks, particularly the inadvertent injury to vascular structures located in the anterior midline of the neck. Such injuries can cause serious complications, including hematoma, airway obstruction, and even mortality [4]. Previous studies have reported that vascular structures may be present in the midline neck region in a significant proportion of patients, with prevalence estimates reaching up to 41%, highlighting a clinically significant risk during blind landmark-based procedures [5].

Ultrasound (US) has emerged as a non-invasive and portable imaging modality that enables real-time visualization of neck anatomy, including tracheal landmarks and critical vasculature [5]. Its use can enhance the safety and accuracy of tracheotomy and cricothyrotomy by helping clinicians identify individual anatomical variations and avoid vascular injury. Despite this, US guidance is not yet routinely incorporated into airway management protocols [6].

This study aims to investigate the prevalence of midline vascular structures in the anterior neck region relevant to tracheotomy and cricothyrotomy and to evaluate the role of US in reducing procedural risks. By enhancing anatomical understanding and procedural safety, this research supports the advancement of airway management practices toward greater patient safety.

## Materials and methods

This cross-sectional observational US study was conducted on 80 healthy adult volunteers. All participants had no prior history of tracheotomy or cricothyrotomy and no known neck pathology. Volunteers were recruited from university staff and students through convenience sampling. Individuals with a history of neck trauma, surgery, tumors, vascular malformations, or other neck-related disorders were excluded to minimize anatomical variability. Exclusion criteria were confirmed using self-report and brief physical examination.

US equipment and operator

US examinations were carried out using a GE LOGIQ e ultrasound system (GE Healthcare, Chicago, IL) equipped with a 12L-RS linear transducer (5-12 MHz). All scans were performed by a physician with more than five years’ experience in clinical US, ensuring procedural consistency and reducing inter-observer variability.

US procedure

Each volunteer was positioned supine with the head in slight extension and the upper body elevated to approximately 30-45 degrees, simulating a clinically relevant airway access posture used in tracheotomy and cricothyrotomy procedures. A transverse (short-axis) US scan was performed using the linear probe placed transversely across the neck at three specific anatomical levels:

1. Inferior border of the thyroid cartilage

2. Superior border of the cricoid cartilage

3. Inferior edge of the thyroid isthmus.

**Figure 1:**
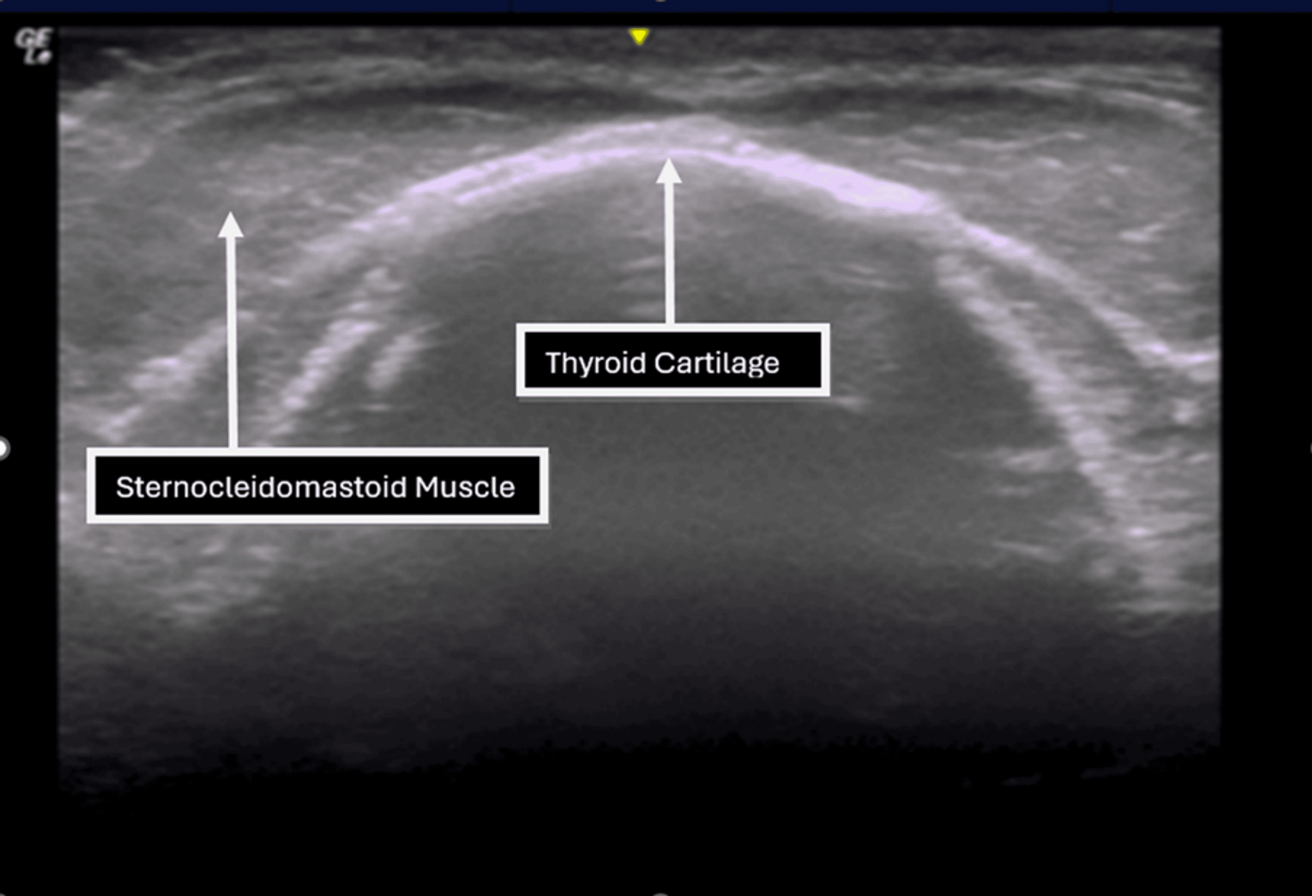
Transverse ultrasound image at the level of the inferior border of the thyroid cartilage.

**Figure 2:**
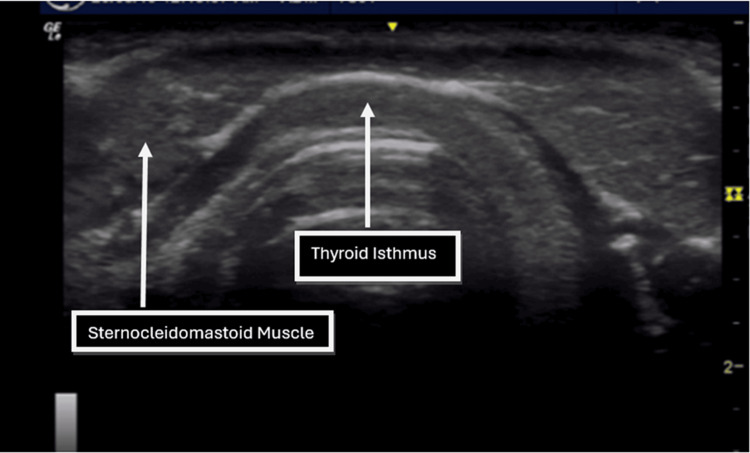
Transverse ultrasound image at the level of the inferior border of the thyroid isthmus.

**Figure 3:**
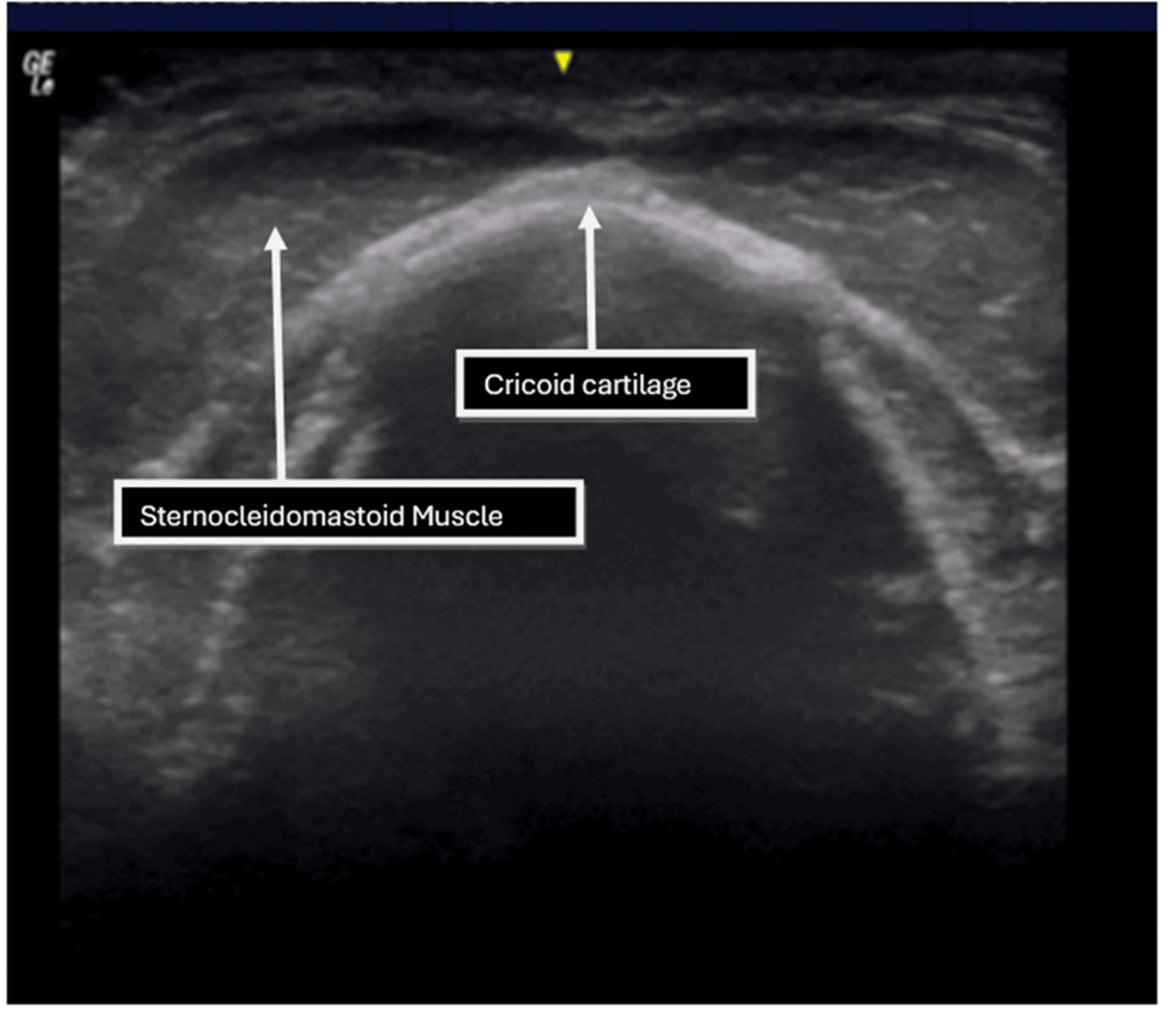
Transverse ultrasound image at the level of the superior border of the cricoid cartilage.

At each level, the probe was adjusted for optimal image acquisition. The presence or absence of midline vascular structures was noted. Vessels were defined as compressible (veins) or pulsatile/non-compressible (arteries), though detailed vessel classification was not consistently possible across all participants. All US images were reviewed in real time during the scan and again post-scan to confirm findings. The primary outcome was the presence or absence of midline vascular structures at each of the three predefined anatomical levels (Figures 1-3).

Data analysis

Data were recorded in a standardized spreadsheet, and statistical analysis was performed using SPSS version 26.0 (IBM Corp., Armonk, NY). Descriptive statistics were used to calculate the prevalence of midline vascular structures at each level, expressed as percentages and with corresponding 95% confidence intervals (CIs).

Comparative analyses were conducted to evaluate the relationship between the presence of midline vasculature and demographic variables (sex, BMI, and ethnicity). Chi-square tests were applied for categorical variables, and logistic regression was used to identify any independent associations. The prevalence of midline vascular structures at each anatomical level was reported with corresponding 95% CIs using the exact (Clopper-Pearson) method. A p-value <0.05 was considered statistically significant. A priori sample size calculation was not performed; the study size was based on feasibility and is acknowledged as a limitation.

## Results

**Figure 4:**
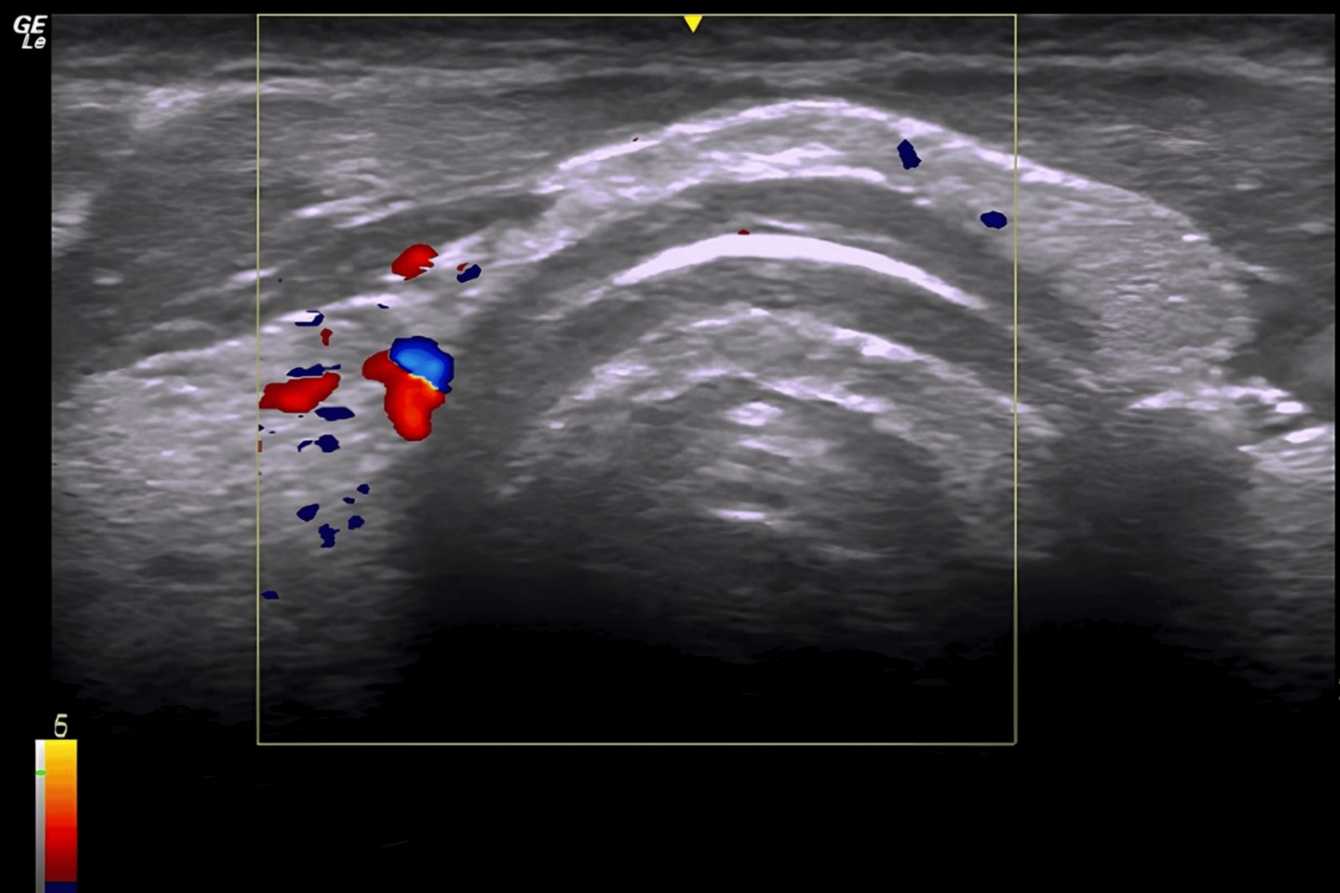
Transverse ultrasound image showing vessels around thyroid gland.

A total of 80 healthy volunteers were scanned (46 male volunteers and 34 female volunteers), with an age range of 18-49 years (mean age: 25.4 ± 7.2). US imaging revealed the presence of midline vessels in 23 participants (28.7%), as shown in Figure 4, whereas 57 (71.3%) participants did not have midline vessels.

US imaging also revealed variable prevalence of midline vascular structures at different anatomical landmarks in the cervical region, as seen in Table 1. At the level of the inferior edge of the thyroid isthmus, midline vascular structures were identified in 18 participants (22.5%). At the level of the superior border of the cricoid cartilage, midline vasculature was seen in three participants (3.8%). At the inferior border of the thyroid cartilage, vascular presence was lowest, observed in only two participants (2.5%).

**Table 1:** Prevalence of midline vascular structures by anatomical landmark.

Anatomical Landmark	Participants With Midline Vessels (n)	Total Participants (N)	Prevalence (%)	95% CI (Clopper-Pearson Exact) (%)
Inferior edge of the thyroid isthmus	18	80	22.5	13.7-32.8
Superior border of the cricoid cartilage	3	80	3.8	0.8-10.7
Inferior border of the thyroid cartilage	2	80	2.5	0.3-8.7

Importantly, in this study, the presence of midline vessels showed no significant association with gender (χ²(1) = 0.15, p = 0.699) or ethnicity (χ²(3) = 0.34, p = 0.953), suggesting that these vascular variations occur independently of demographic factors, as shown in Table 2. There was no statistically significant correlation between the presence of midline vasculature and sex, BMI, or ethnicity (p > 0.05 across comparisons using chi-square or Fisher’s exact test as appropriate). These results suggest that vascular variation in this region is not easily predictable based on patient demographics, as shown in Table 3.

**Table 2:** Association between midline vascular structures and demographic variables.

Variable		Vessels Present, n (%)	Vessels Absent, n (%)	χ² (df)	p-Value
Gender				0.150 (1)	0.699
	Male	14 (30.4)	32 (69.6)		
	Female	9 (26.5)	25 (73.5)		
Ethnicity				0.338 (3)	0.953
	White (n=53)	15 (28.3)	38 (71.7)		
	Black (n=11)	3 (27.3)	8 (72.7)		
	Asian (n=11)	3 (27.3)	8 (72.7)		
	Mixed (n=5)	2 (40.0)	3 (60.0)		

**Table 3:** Study participants' demographics.

Demographics		n (%)/Mean
Age (years)		25.4 ± 7.2 (18-49)
Gender	Male	46 (57.5%)
	Female	34 (42.5%)
Ethnicity	White	53 (66.3%)
	Black	11 (13.8%)
	Asian	11 (13.8%)
	Mixed	5 (6.3%)
BMI (kg/m²)		22.7 ± 3.3 (17-29)

## Discussion

The study offers critical insights into the anatomical variations of anterior neck vasculature and their implications for airway management procedures such as tracheotomy and cricothyrotomy. Among the study participants, vascular prevalence at the cricothyrotomy level was markedly lower (2.5-3.8%), supporting its relative safety as an emergency airway procedure. Our findings are also consistent with previous studies. For example, cadaveric studies have identified midline vasculature, particularly the inferior thyroid vein, in up to 97.5% of specimens [7] while live imaging studies reported lower rates ranging from 0.16% to 48.8%, because smaller venous branches may have been missed [8,9].

This presence of midline vessels is clinically relevant and highlights the risk of vascular injury that may often be overlooked when these procedures are performed without image guidance. Common risks associated with these procedures include hemorrhage, hematoma, and airway compromise. These risks may be magnified in patients with obesity, neck tumors, or distorted anatomy, where surface landmarks are unreliable [10]. Importantly, national and international guidelines, including American Society of Anesthesiologists (ASA) and Difficult Airway Society (DAS) recommendations, do not currently mandate routine pre-procedural US [11], which highlights a gap between emerging evidence and practice. One possible reason why ASA and DAS do not mandate pre-procedural US could possibly be because of emergency timing, limited operator availability, or lack of access to equipment in resource-poor settings.

US offers the advantages of portability, real-time imaging, and non-invasiveness. By detecting patient-specific anatomical variations it provides a low-cost means to prevent vascular complications and enhance procedural safety. Its use enables precise identification of the trachea, cricothyroid membrane, and adjacent vasculature, thereby significantly reducing the risk of inadvertent injury [11].

Beyond the technical and safety benefits, the US also holds educational value. Its integration into medical training enhances anatomical understanding, improves procedural confidence, and fosters early competence in airway management techniques [12]. Several studies have shown that exposure to the US at the undergraduate or early postgraduate level leads to improved clinical outcomes and decision-making, especially in high-stakes environments [13,14]. Importantly, in this study, the presence of midline vessels showed no significant association with gender (χ²(1) = 0.15, p = 0.699) or ethnicity (χ²(3) = 0.34, p = 0.953), suggesting that these vascular variations occur independently of demographic factors.

The small sample size limits the generalizability of the findings. In addition, the use of convenience sampling from a single center may limit representativeness. Finally, resource constraints, including limited access to multiple operators and equipment, prevented assessment of inter-operator variability and restricted the scope of data collection.

Future research should involve larger, multicenter cohorts with heterogeneous populations and include inter-operator reliability assessments. Studies conducted in emergency settings would also help determine whether the US can be feasibly integrated into time-critical airway management.

## Conclusions

This study highlights the clinically significant presence of midline vascular structures in the anterior neck, particularly at the level of the thyroid isthmus, in healthy individuals, as detected via high-resolution US. These findings highlight the potential risk of vascular injury during tracheotomy procedures performed using traditional landmark-based methods. In contrast, the incidence of vascular structures at levels associated with cricothyrotomy was notably lower, supporting its relative safety in emergency airway management. US, as a non-invasive, portable, and real-time imaging modality, offers clear advantages in both elective and emergent airway procedures. Its ability to identify individual anatomical variations, particularly vascular anomalies, makes it an essential adjunct to safe airway access. In conclusion, the integration of point-of-care US into tracheotomy and cricothyrotomy practice represents a critical step forward in improving safety and reducing avoidable complications in airway management.
